# Influenza A Viruses from Wild Birds in Guatemala Belong to the North American Lineage

**DOI:** 10.1371/journal.pone.0032873

**Published:** 2012-03-13

**Authors:** Ana S. González-Reiche, María E. Morales-Betoulle, Danilo Alvarez, Jean-Luc Betoulle, Maria L. Müller, Silvia M. Sosa, Daniel R. Perez

**Affiliations:** 1 Department of Veterinary Medicine, University of Maryland College Park, and Virginia-Maryland Regional College of Veterinary Medicine, College Park, Maryland, United States of America; 2 Laboratorio de Ecología de Arbovirus y Virus Zoonóticos, Centro de Estudios en Salud, Universidad del Valle de Guatemala (CES-UVG), Guatemala City, Guatemala; 3 Fundación Para el Ecodesarrollo y la Conservación (FUNDAECO), Guatemala City, Guatemala; University of Ottawa, Canada

## Abstract

The role wild bird species play in the transmission and ecology of avian influenza virus (AIV) is well established; however, there are significant gaps in our understanding of the worldwide distribution of these viruses, specifically about the prevalence and/or significance of AIV in Central and South America. As part of an assessment of the ecology of AIV in Guatemala, we conducted active surveillance in wild birds on the Pacific and Atlantic coasts. Cloacal and tracheal swab samples taken from resident and migratory wild birds were collected from February 2007 to January 2010.1913 samples were collected and virus was detected by real time RT-PCR (rRT-PCR) in 28 swab samples from ducks (*Anas discors*). Virus isolation was attempted for these positive samples, and 15 isolates were obtained from the migratory duck species Blue-winged teal. The subtypes identified included H7N9, H11N2, H3N8, H5N3, H8N4, and H5N4. Phylogenetic analysis of the viral sequences revealed that AIV isolates are highly similar to viruses from the North American lineage suggesting that bird migration dictates the ecology of these viruses in the Guatemalan bird population.

## Introduction

The role of wild birds in the transmission of AIVs has become highly significant with the introduction and spread of Highly Pathogenic Avian Influenza Viruses (HPAIV) of the H5N1 subtype into different countries in Asia, Europe, and Africa [1], [2], [3], [4]. It is generally accepted that aquatic wild birds are the primary reservoirs of AIVs as evidenced by the fact that most of the different possible combinations of HA and NA subtypes (e.g. H4N2) have been found in these animals [4], [5]. AIVs have been isolated from over 100 species of wild birds belonging to 12 different orders, mainly Anseriformes and Charadriiformes [6], [7]. The virus has also been reported at low prevalence in small terrestrial birds (e.g. Passerines) ranging from 0.9% to 6.6% [8], [9], [10] and it has been proposed that such species can act as bridges between the wild aquatic and domestic birds because they co-exist with both ecosystems [11], [12], [13], [14], [15], [16]. Several surveillance studies have provided insight into the evolution of AIVs and its relationship with wild bird behavior [17], [18], [19], [20]. Information regarding intercontinental exchange of viruses and genetic reassortment between Eurasian and North American viruses has been reported [21], [22], [23]; however, little is known about the exchange of genetic material between viruses in the Americas, particularly between the Northern and Southern hemispheres. In Central America, the presence of AIV was confirmed with the isolation of the low pathogenic avian influenza virus (LPAIV) of the H5N2 subtype from poultry in 2000 in Guatemala and 2001 in El Salvador. Genetic characterization of the H5N2 isolates revealed that the virus was most likely introduced from Mexico [24]. Vaccination against H5N2 in Guatemala has been used as the primary control strategy [25]. The circulation of other AIV subtypes of poultry in Guatemala, and elsewhere in Central America, has not been reported, although it must be noted that there has been limited surveillance.

Guatemala is located on a geographic bottleneck (the Central American Isthmus) that funnels millions of migrating birds from several North American flyways (Mississippi, Pacific and Atlantic American) through a narrow area. The tropical habitats of Central America constitute a terrestrial bridge between North and South America for well over 120 species of migratory birds [26]. The forests and wetlands of tropical areas provide shelter and stopover habitats for several species of terrestrial and aquatic migratory birds [27], [28]. As it has been hypothesized, these sites could be important for AIV transmission and reassortment between different bird species and from different migration flyways [5]. To date, there is very little information regarding the circulation and ecology of AIV in Central America and only recently information about AIV in wild birds from South America has been reported [19], [29], [30], [31]. Thus the role of specific bird species in the spread of AIVs throughout these regions remains unclear [32], [33], [34].

In this study we conducted surveillance of AIV in wild birds in several sites along the Pacific and Atlantic coasts of Guatemala. Resident and migratory wild birds associated with aquatic habitats were sampled to detect the presence of AIV. The aim of the study was to provide an initial assessment of the presence and ecology of avian influenza viruses in Guatemala and serve as a platform for the early detection of the introduction of HPAIV strains (e.g. H5N1) from wild birds.

## Results

**Figure 1:**
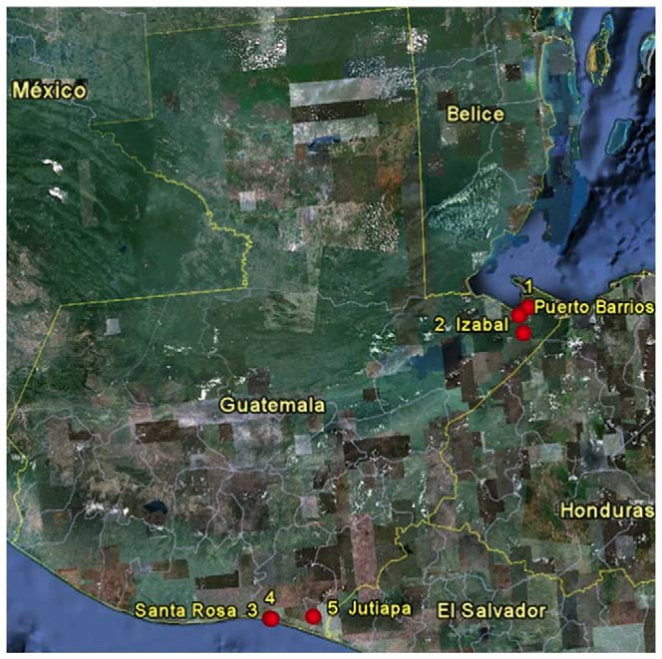
Location of sample collection sites in the Atlantic (1 and 2) and the Pacific (3, 4, and 5) coasts of Guatemala. Latitude and longitude of surveillance sites are provided in Table 1.

From February 2007 to January 2010, AIV surveillance was conducted in five locations in Guatemala, two sites on the Atlantic coast within the state of Izabal (villages of Puerto Barrios and Machacas del Mar), and three sites on the Pacific coast wetlands, in the states of Santa Rosa (villages of Monterrico and El Pumpo) and Jutiapa (village of Pasaco) (Table 1, Fig. 1). 1913 tracheal and cloacal swabs were collected from 969 birds from 78 different species, representing 22 different families and 11 different orders (Table S1). 50.4% (489/969) were resident and 49.6% (480/969) were migratory bird species. Samples were produced from sport-hunter killed aquatic birds or from shorebird species of the orders Anseriformes (n = 239), Gruiformes (n = 4), and Pelecaniformes (n = 1). The remaining samples (n = 725) were collected from live captured terrestrial birds that were under study as part of West Nile virus surveillance (Morales-Betoulle, unpublished data).

**Table 1:** Sites for avian influenza surveillance in wild birds, Guatemala, 2007–2010.

Location	State	Site	Latitude (N)	Longitude (W)	Collection method*	Season
Atlantic Coast	Izabal	Machacas del Mar	15°45′48.10″	88°31′48.60″	A	2007–2009
		Puerto Barrios	15°43′0.00″	88°35′60.00″	A	2007–2009
Pacific Coast	Santa Rosa	Monterrico	13°53′39.00″	90°28′48.00″	A/K	2007–2010
		El Pumpo	13°53′51.80″	90°29′33.20″	K	2007–2010
	Jutiapa	Pasaco	13°53′8.60″	90°11′45.30″	K	2009

A = Active surveillance (mist nets, live captured birds), K = Hunter-killed.

The RNA extracts from collected samples were tested by rRT-PCR assay for the detection of type A influenza virus [35]. From these, 2 tracheal and 30 cloacal swabs tested positive (Table S1) corresponding to 28 dabbling ducks (Blue-winged Teal), and 2 resident terrestrial birds: a Golden-fronted woodpecker (Piciformes) and a Brown-crested flycatcher (Passeriformes). In 2ducks, both cloacal and traqueal swabs were positive for AIV. The presence of AIV was detected every year of the study. The overall percentage of rRT-PCR positive samples (tracheal and cloacal swabs) was 1.67%.

To determine factors associated with detection of AIV in wild bird samples, rRT-PCR results were analyzed by habitat (aquatic vs. terrestrial). Detection of AIV was significantly higher in aquatic birds (11.2%) when compared to terrestrial birds (0.3%) (p<0.0001). For the aquatic birds, positive results were obtained for the Blue-winged Teals, thus comparisons for this species were done by specimen's age (juvenile vs. adult), sex (female vs. male).AIV detection rate was similar for both juvenile (12.5%, n = 27) and adults (12.8%, n = 129), and for females (14.9%, n = 87) and males (10.1%, n = 129). No significant differences were observed between these categories.

In Blue-winged teals, the percentage of rRT-PCR positive samples varied between sampling seasons, 10.0% (2006–07), 7.3% (2007–08), 5.2% (2008–09) and26.6% (2009–10). The number of sampled aquatic birds ranged between 61 to 96 birds per season, with the exception of the 2006–07 season in which only 10 samples were obtained. The proportion of positive samples detected in the 2009–10 season was significantly higher (p<0.0009) compared to the previous seasons. To investigate if AIV prevalence varied during the migratory season, we compared AIV rRT-PCR detection frequencies at the beginning and the end of the seasons. The proportion of AIV positives obtained from October through December (Southern migration) compared from January to March (Northern migration) was 16.7% and 9.7%, respectively; however, the difference was not statistically significant.

Virus isolation was attempted from positive rRT-PCR samples, in 9-day-old SPF embryonated chicken eggs (ECE); 15 viruses were obtained from either the first (9 viruses), second (5 viruses) and third passage (1 virus). After 3 blind passages, no viable viruses were detected in the remaining samples. To identify the subtype of rRT-PCR positives from which viruses could not be isolated, direct sequencing was attempted in cDNA generated from initial RNA extracts; however, no other subtypes could be identified by this method. The percentage of recovered viruses from rRT-PCR positive samples by isolation in ECE was 46.9%. The viruses were isolated only from duck samples of the species *Anas discors* (Blue-winged Teal) obtained from Santa Rosa (2008, 2010) and Jutiapa (2009). After PCR amplification and sequencing of cDNA, the viruses were classified as H7N9 (n = 2), H11N2 (n = 3), H3N8 (n = 1), H8N4 (n = 5), H5N3 (n = 2), H5N4 (n = 2) representing 5 HA and 5 NA different subtypes (Table 2). The two H5N4 isolates were found to contain also N3 NA consensus gene sequences, suggesting a mixed infection in these two samples. Sequencing results of complete virus genomes indicated that these viruses encode for the 11 protein genes known for influenza A viruses, including the 87–90 amino acid protein PB1-F2. The H7 and H5 viruses carry the typical low pathogenic cleavage sites (PENPKTRGLF and PQRETRGLF respectively) [36]. The overall percentage of AIV detection in Blue-winged Teals (n = 234) based on virus isolation was 6.41%. No rRT-PCR positives or virus isolates were obtained from other aquatic bird species including the northern shoveler (*Anas clypeata*, n = 2), ring-neck duck (*Aythya collaris*, n = 2) and black-bellied whistling duck (*Dendrocygna autumnalis*, n = 1).

**Table 2:** Positive species for influenza type A by rRT-PCR and viral isolates obtained in this study.

Season	Location	Species	# Birds sampled	Positives (%)		Virus subtypes
				rRT-PCR*	VI	
2006–2007	Santa Rosa	*Anas discors*	10	1(10)	-	N/D
2007–2008	Santa Rosa	*Anas discors*	96	7(7.3)	2(2.1)	H7N9
2008–2009	Santa Rosa	*Anas discors*	61	3(4.9)	-	N/D
	Izabal	*Melanerpes aurifrons*	21	1(4.8)	-	N/D
		*Myiarchus tyrannulus*	1	1(100)	-	N/D
2009–2010	Jutiapa	*Anas discors*	20	4(20)	3(1.5)	H11N2
	Santa Rosa	*Anas discors*	47	13(27.7)	10(21.3)	H8N4 (5), H5N3 (2), H5N4 (2), H3N8 (1)
**Total**				**30**	**15(1.6)**	**6**

* Percentage of positive samples obtained by real-time RT-PCR (rRT-PCR) and Virus Isolation (VI) based on the total number of sampled birds.

N/D: Non-Determined.

**Figure 2:**
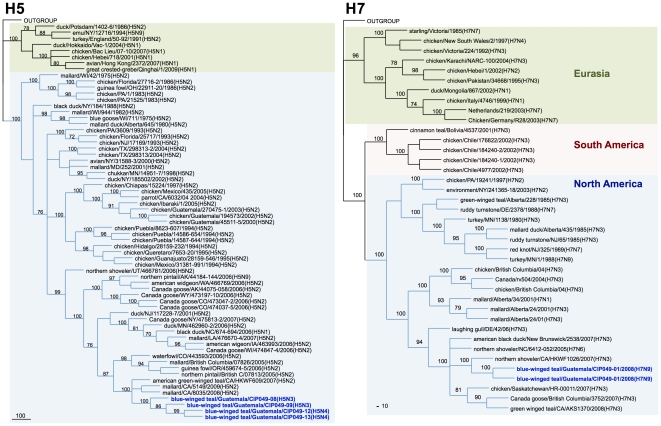
Phylogenetic trees for H5 and H7 HA genes. Trees were generated in PAUP 4.0b10 using the Neighbor-Joining method with 1000 bootstrap replicates (bootstrap values above 70% are shown). Scale bar on the bottom-left indicates number of nucleotide substitutions per site.

**Figure 3:**
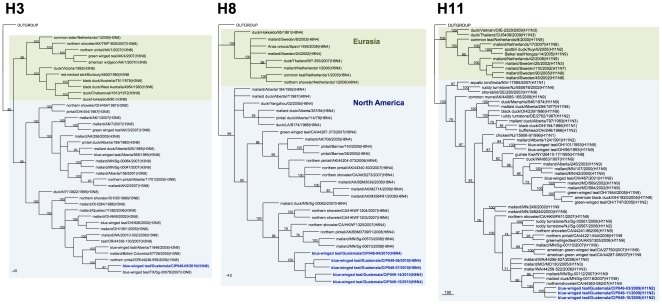
Phylogenetic trees for H3, H8 and H11 HA genes. Trees were generated in PAUP 4.0b10 using the Neighbor-Joining method with 1000 bootstrap replicates (bootstrap values above 70% are shown). Scale bar on the bottom-left indicates number of nucleotide substitutions per site.

**Figure 4:**
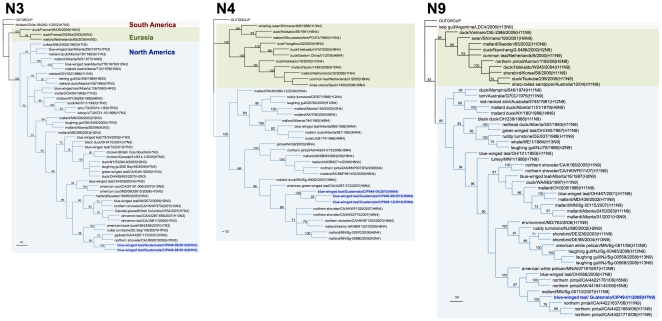
Phylogenetic trees for N3, N4, and N9 NA genes. Trees were generated in PAUP 4.0b10 using the Neighbor-Joining method with 1000 bootstrap replicates (bootstrap values above 70% are shown). Scale bar on the bottom-left indicates number of nucleotide substitutions per site.

**Figure 5:**
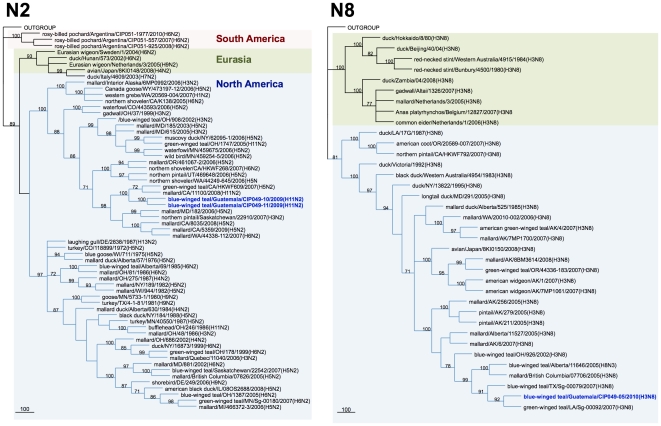
Phylogenetic trees for N2 and N8 NA genes. Trees were generated in PAUP 4.0b10 using Neighbor-Joining method with 1000 bootstrap replicates (bootstrap values above 70% are shown). Scale bar on the bottom-left indicates number of nucleotide substitutions per site.

**Figure 6:**
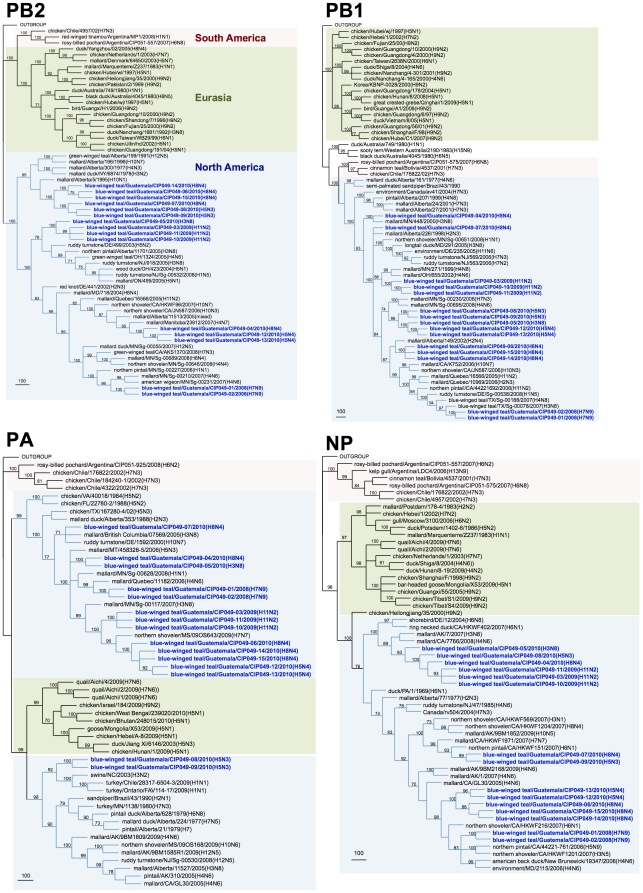
Phylogenetic trees for internal gene segments PB2, PB1, and PA. All trees were generated in PAUP 4.0b10 using Neighbor-Joining method with 1000 bootstrap replicates (bootstrap values above 70% are shown). Scale bar on the bottom-left indicates number of nucleotide substitutions per site.

**Figure 7:**
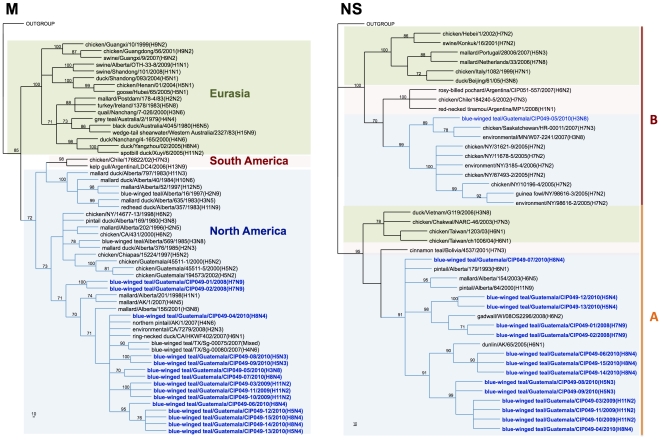
Phylogenetic trees for internal gene segments NP, M and NS. All trees were generated in PAUP 4.0b10 using Neighbor-Joining method with 1000 bootstrap replicates (bootstrap values above 70% are shown). Scale bar on the bottom-left indicates number of nucleotide substitutions per site.

Genetic similarity among the internal gene segments of the Guatemalan isolates ranged from 72.5% for the non-structural gene (NS) to 100% for the matrix (M) gene. For phylogenetic analysis, identical sequences were excluded and only one representative of each sequence was used. Sequence comparison by BLAST searches and phylogenetic analysis revealed that surface glycoprotein genes of the Guatemalan isolates share sequence identity and cluster with segments of AIV strains isolated from waterfowl of North American (Fig. 2, 3, 4, 5). The HA and NA gene segments were found to be closely related to strains isolated along the Mississippi (H3N8 subtype) and Pacific American flyways (other subtypes). As expected, the internal gene segments were also phylogenetically related to North America strains. A single isolate (H3N8) carry a NS gene segment corresponding to allele B, whereas the rest carry an allele A (Fig. 6, 7).

**Figure 8:**
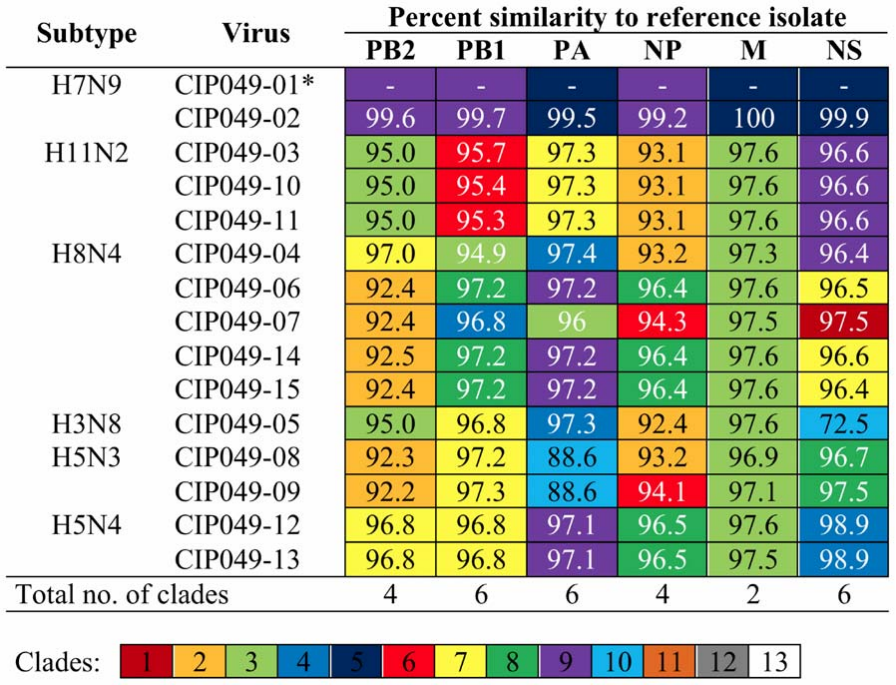
Genome constellations of AIVs obtained from wild birds in Guatemala. Nucleotide percent similarities are shown. The different colors represent different clades supported by bootstrap values >70%. *Isolate CIP049-01 was used as reference to estimate sequence percent similarities.

The Guatemalan isolates were classified among distinct clades of the North America lineage. Clade identification was supported by bootstrap values of >70% [37]. 8 clades were identified for segment M, 9 for segments PB2, PB1 and NP, and 13 clades were identified for PA and NS (including alleles A and B) segments. An identification number from 1 to “n” (n = 8, 9 or 13) was assigned to each clade of each gene segment to allow inference regarding genetic similarities and reassortment events that may have occurred within a given viral subtype. Based on this analysis, 9 unique genome constellations were identified among the 15 isolates (Fig. 8), although only on the H8N4 viruses (n = 5) there was evidence of 4 different gene constellations, indicative of reassortment events. The H5N3 viruses (n = 2) belonged to the same constellation except for the NP gene. For the other strains – H7N9, H11N2, and H5N4 – each subtype contained its own constellation of segments. These results suggest multiple introductions and reassortment of AIVs in wild birds in Guatemala.

## Discussion

In Central America, the ecology of AIVs is not well understood. In order to collect surveillance data from this understudied region, several variables were considered to evaluate the presence/absence, diversity, and seasonality of AIVs. Such variables included type of sample(s), target species and study sites. Based on these variables, hunter-harvested waterfowl and terrestrial bird species that were under surveillance for other zoonotic diseases were chosen as target population. Sampling hunter-harvested waterfowl is a convenient method to collect bird samples during the migration season. In addition, sampling of terrestrial birds associated with coastal and aquatic habitats may provide more insights into the role of these species as vectors between aquatic and poultry species.

In this study, AIV was detected by rRT-PCR in tracheal and cloacal swabs from migratory ducks from all the study sites where samples were collected. The majority of rRT-PCR positive samples, and consequently all virus isolates, were obtained from the wild duck species *Anas discors* (Blue-winged teal) sampled in wintering seasons (from late October to early March). The hypothesis that dabbling ducks play an important role in maintaining AIV transmission in nature by feeding on the water surface is supported by surveillance studies in wild ducks and environmental sampling [34], [38]. In our case, the overall proportion of rRT-PCR positive birds obtained in this study is in agreement with findings reported for other geographical regions. Specifically, we found rRT-PCR positive samples in 11.2% of waterfowl. The reported prevalence of LPAI in sampled waterfowl ranges between 0.03 to 22.2% [18], [39], [40]. In the case of waterfowl, this prevalence value tends to be higher after breeding in temperate zones and peaks between late summer and early fall before migration occurs [7]. Our results are in agreement with other studies, particularly for Blue-winged teals, reported prevalence estimates ranges from 6.6% to 10.9% in temperate zones [41] and from 4.2% up to 22% in wintering grounds in North America[42], [43], [44]. In our study, the proportion of AIV Blue-winged teals detected in the early months of the migratory season was apparently higher than the proportion of positives at the end of the season. Although the difference was not statistically significant this finding could support the previous observations that the AIV prevalence decreases during migration [18]. However, approximately 10% of the ducks were infected close to the end of the migration period, suggesting that these birds could still carry a significant amount of AIV prior to returning to the temperate zones. Our findings are further supported by other studies in wintering areas in the United States, where late winter infection in this particular species at relatively high prevalence (>10%) has been observed [42].

When the prevalence values for AIV in Blue-winged teals were compared by age groups (juvenile or adult), no significant differences were observed, which is in contrast to what has been reported elsewhere where juvenile birds tend to harbor higher prevalence of infection than adults [18], [44], [45]. This discrepancy may be explained by the limited sample size of our study (n = 27 for juvenile and n = 129 for adult teals), which may have resulted in low power in the statistical analyses. The Blue-winged teal is a dabbling duck species that performs a long-distance migration to Central America, the Caribbean, and some areas of South America. It is one of the first species to migrate south and one of the last to return to the north [46], [47]. In Guatemala, the blue-wing teal is one of the most abundant of the 16 Anseriformes species reported, with daily counts as high as 8,000 individuals during the last months of the wintering season [48]. Their early migration to the south together with other behavioral and ecological factors may influence the role of blue winged teals as reservoirs for AIV [49], [50]. The impact that long-distance traveling may have on their immunological status [50] may also contribute to explain the fact that adults in this study were found infected in a similar proportion to juvenile birds. This could be important as this may increase the number of available reservoirs for virus infection at the migration sites.

An increase in virus detection was observed in the 2009–10 sampling season, in which one sampling site was added (Jutiapa). However, the majority of positive samples came from the site in Santa Rosa, where no significant changes occurred in the number of collected samples compared to the previous years. As it has been described previously, the increase in virus detection/prevalence could be related to a seasonal pattern followed by some influenza viruses [18]. Only long-term surveillance together with the implementation of more systematic sampling methodologies will provide more and better surveillance data to support this finding.

In this study, a wide diversity of virus subtypes was observed in Blue-winged teals in3 out of the 4 sampling seasons. Interestingly, even though the AIV subtypes isolated in Guatemala have been isolated in North America with the same HA/NA combinations, most of them have been isolated only sporadically or at low frequencies. Some of these subtypes, such as the H5N4 has been reported only once [18]. Here the most detected subtype combination was H8N4 (5 out of 15 isolates). In contrast, other subtypes more prevalent in North America, such as H4 and H6, were not detected in our study [49]. It is important to note that the number of sampled species, seasonal variation as well as the adaptation of the viruses to different environmental conditions may influence the diversity and prevalence of isolated subtypes. Despite the limited number of sampled birds and isolates obtained, the fact that these “low prevalent” subtypes for North America were most frequently detected in Guatemala warns of the possibility that stopover habitats could function as repositories for maintenance of subtypes and genetic diversity. Our findings are supported by other studies at wintering areas in Texas, where the subtype diversity was mainly represented by non-frequently occurring subtypes, including the H8 [42].

Both H5 and H7 subtypes were isolated during this study. Although H5N1 and H7N3 subtypes are of most interest for their association with emergence of HP strains [37], other combinations such as the ones isolated here (e.g. H5N3) have been related to outbreaks of LP AIV in turkey farms and other poultry species [51]. Further characterization of the pathogenicity of the viruses in chickens and other avian models in the laboratory could help address the significance and potential impact on poultry population of the circulation of H5 and H7 subtypes in the region.

As revealed by phylogenetic analysis the Guatemalan isolates are more closely related to recent isolates from the Mississippi and Pacific American flyways. Although there is limited sequence data and information of AIV viruses circulating in wild bird populations of adjacent territories (Mexico and other Central and South American countries), this observation bolster the possibility that the viruses are being introduced or more likely in constant exchange by migratory birds coming from the North.

For the internal genes (PB2, PB1, PA, NP and NS) the nucleotide sequences exhibited higher diversity as evidenced by the number of genome constellations. This observation suggests that there have been multiple AIV introductions into the coastal sites in Guatemala. In addition, the finding of two H5 strains with similar HA genes but with different NA subtypes (N3 and N4), represents potential evidence for reassortment between viruses at the site of sample collection. These observations are consistent with other studies where frequent reassortment has been found to occur between viruses recovered from the same sites [37] over several years [21], [52], and supports the idea of independent reassortment between gene segments and continuous virus introduction and exchange by wild birds.

Recently, several AIV isolates from North and South American countries including Mexico, Argentina, Chile, and Bolivia have been described [19], [29], [30], [53] . In addition, there is evidence that South American viruses constitute a genetic subgroup distinct from other influenza viruses [54]. The occurrence and frequency of reassortment between these two lineages or genetic groups and/or the exchange of virus between North and South American territories remains unknown. The high frequency of detection and genetic diversity reflects multiple AIV introductions from numerous waterfowl populations from North America occurring each year. Virus exchange between migration flyways at wintering grounds could result in virus reassortment upon bird's arrival to temperate zones. In the case of Blue-winged teals, it is not entirely clear whether conspecific populations breed in the tropics, as it has been observed that some small groups of ducks do not return north after the fall migration [55], [56]. As competent reservoirs, these conspecific populations could play an important role in not only introducing AIV subtypes into the tropics, but also transmitting and perpetuating them among local bird populations during the non-migration seasons. Further sampling during the non-migration season is essential to confirm this hypothesis.

In addition to AIV detection and isolation from wild aquatic birds, two positive samples were obtained from a single flycatcher and a woodpecker, both of which are non-migratory species. However, virus presence in these samples could not be confirmed by virus isolation or direct sequencing of cDNA. The significance of AIV RNA detection in two non-aquatic resident species needs further investigation, including virological and serological surveillance in these species could provide more insights on the importance of these birds as reservoirs of AIV.

The tropical wetlands and forest of Guatemala are regions with great diversity of avian species [57]. The impact of AIV circulation in a high species diversity ecosystem such as the neotropics needs further study. Only prolonged research of influenza viruses in Central America and other South American territories will provide insight into the seasonality, molecular evolution and exchange of genetic material between South and North American viruses carried by avian hosts. Moreover, the study sites are located near rural communities with scarce resources where the habitants often depend on poultry farming and live in close contact with their domestic and free-ranged animals, as well as wild animals. Summed to this, cultural background and limited resources hinder the establishment of adequate biosecurity practices. In this context, considering the geographic spread of HPAI H5N1 [58], and the frequency of outbreaks of H5 and H7 (LPAI and HPAI) viruses in different regions worldwide [34], [54], [59], [60], [61], [62], [63], [64], [65], [66], [67], [68], the presence of H7 and H5 viruses in wild birds crossing into Central America represents a threat to domestic fowl that cannot be ignored.

In summary, 15 isolates of LPAI from 6 different subtypes including H5 and H7 were recovered from wild aquatic birds in Guatemala. Most of the isolated subtypes constitute a group of viruses that have been sporadically found in other geographical regions. Phylogenetic analysis suggests that these viruses are genetically similar to North American strains. Our findings provide the first description of LPAI isolated from wild birds in Central America, and provide clear evidence of frequent introduction and exchange of AIV in the Neotropical ecosystems by migratory birds. These findings highlight the importance of continued surveillance efforts of AIV not only in wild but also domestic birds in the central and southern western hemisphere.

## Methods

### Ethics statement

Collection of bird samples were approved by the Institutional Animal Use and Care Committee of the Universidad del Valle de Guatemala and reviewed and approved by the Institutional Animal Use and Care Committee of the University of Maryland, College Park under protocol number R-08-10. Sampling of hunter-killed birds were exempt of animal use and care regulations.

We conducted AIV surveillance in different sites of Guatemala in the Atlantic and the Pacific coast. For all sampling activities official permits were approved by the Center for Conservation Studies (CECON) and the National Council of Protected Areas (CONAP). The Ministry of Agriculture of Guatemala (MAGA) approved the study.

### Sample collection

Samples were collected by trained veterinarians and technicians from the Center of Health Studies of University del Valle de Guatemala (CHS-UVG). Tracheal and cloacal swab specimens were obtained from hunter-killed ducks and mist net captured birds. Captures with mist nets and sampling were conducted at the study sites of Monterrico, Machacas del Mar and Puerto Barrios every 5–6 months, from October 2007 to July 2009. For mist-net captured birds, cloacal and tracheal swabs were collected from all animals, except for small birds in which case only cloacal swabs were collected. In addition to mist net captures, sport hunters were contacted for sampling of aquatic birds immediately after hunting. Samples from killed birds were obtained during the migratory seasons of 2007, 2008, and 2009 in Monterrico, El Pumpo and Pasaco villages. Prior to specimen collection, trained ornithologists and technicians identified birds by species, sex, and age (adult, juvenile, hatch year or after hatch year). After specimen collection, swabs were placed into 1–1.5 mL of freshly prepared viral transport medium (VTM, Medium 199 with Hanks balanced salt solution, 2 mM L-glutamine, 0.5% bovine serum albumin, 0.35 g/liter sodium bicarbonate) with antibiotics and antimycotics (2×10^6^ IU/L Penicillin, 200 mg/L Streptomycin, 2×10^6^ IU/L Polimyxin B, 250 mg/L Gentamycin, 0.5×10^6^ IU/L Nistatin, 60 mg/L Ofloxacine, and 0.2 g/L Sulphamethoxazol) [69]. Specimens were transported to the laboratory on ice, frozen in liquid nitrogen or on dry ice in double sealed plastic bags depending on availability and estimated time of delivery to the laboratory. Samples were then stored at −70°C until processed.

### Type A influenza virus detection by rRT-PCR

For tracheal swabs, RNA was extracted from 200 µL of supernatant with QIAamp viral RNA kit (QIAGEN, Valencia, CA). Extracted RNA was eluted from the QIAgen columns to a final volume of 100 µL of elution buffer and stored at −70°C. For cloacal specimens, RNA was extracted from 250 µL of supernatant with Trizol LS reagent (Invitrogen, Carlsbad, CA) [70]. Extracted RNA was then resuspended in 100 µL of DEPC treated water, and stored at −70°C until tested for molecular detection of influenza viral RNA (vRNA).

Prior to sample testing, a formerly reported rRT-PCR assay for Type A influenza virus detection [35] was optimized at CHS-UVG. For standardization, influenza type A viral RNA was extracted from a clinical sample provided by Dr. A. Estevez (Laboratory of Respiratory Diseases, IEIP, CHS-UVG) and was used as positive control to determine the detection limits of the assay. Transcribed RNA from cloned matrix (M) protein (A/Guinea/fowl/HK/99/H9N2) in pDP2002 was also used as positive control [71]. Plasmid DNA was transcribed with the T7 RiboMAX™ Express Large Scale RNA Production System (Promega, Madison, WI) in accordance with manufacturer's instructions. Clinical sample RNA and transcribed RNA were quantitated by spectrophotometer (GenSpecI, Naka Instruments, Dalian, China).

All rRT-PCR reactions with matrix gene specific primers and probe were carried out using the QuantiTect Probe RT-PCR Kit (QIAGEN, Hilden, Germany) and the ABI 7300 Real-Time PCR System (Applied Biosystems, Foster City, CA). For a 25 µL reaction mixture the following conditions were used: 12.5 µL of kit-supplied 2× RT-PCR master mix, 10 pmol of each primer, 0.3 µM probe, 0.25 µL of kit-supplied enzyme mix, 6.5 U RNase inhibitor and 8 µL of RNA template. Thermal cycling conditions comprised one cycle of reverse transcription at 50°C for 30 min and 94°C for 15 min, followed by 45 cycles of denaturation at 94°C for 1 s and a combined annealing and extension at 60°C for 27 s. Fluorescence signal was obtained at the end of each cycle after the annealing/extension step. After amplification, quantitation data were analyzed with the 7300 System SDS Software v1.4.0 (Applied Biosystems). Positive control RNA was calibrated to a Ct value between 25 and 35 for the diagnostic purposes of the assay [70].

Eight µL of RNA samples extracted from tracheal and cloacal swabs were analyzed by rRT-PCR for Type A influenza virus detection. For each rRT-PCR run, duplicates of calibrated positive control RNA and a water non-template control (NTC) were included. Extracted RNA from cloacal material was analyzed in duplicate and samples with Ct value between 20 and 35 were considered positive. For tracheal swabs, extracted RNA was tested in a single reaction and samples with a Ct value between 20 and 40 were considered presumptive positive and were re-tested for their confirmation. All samples that tested positive for the rRT-PCR assay were processed for viral isolation and molecular characterization.

### Virus isolation and genetic characterization

For viral isolation the swab supernatant of each specimen was filtered and BHI (brain heart infusion media) supplemented with antibiotics and antimycotics was added to a volume of 1.5 mL. A 200 µL volume of this mixture was then inoculated into the allantoic cavity of three, 9-day-old SPF embryonated chicken eggs per sample. Following incubation at 37°C for 72 hours, allantoic fluid was collected and tested for the presence of virus by HA assay and by Flu Detect (Synbiotics, San Diego, CA). After three blind passages in embryonated chicken eggs, samples without virus growth were considered negative for the presence of viable virus.

Viral RNA was extracted from 200 µL of allantoic fluid with RNeasy Mini kit (Qiagen), according to manufacturer instructions. Extracted RNA was eluted in 40 µL of RNase-free water. After cDNA preparation, full-length PCR amplification of the influenza virus segments was performed followed by direct sequencing with the BigDye terminator kit (Applied Biosystems) on ABI 3100 Avant Genetic Analyzer (Applied Biosystems) or ABI 3500 Genetic Analyzer [72]. Segments that could not be sequenced from PCR products were cloned into pCR 2.1 vector using a TA cloning kit (Invitrogen) and were sequenced using vector based M13 primers. At least two sequencing reactions were prepared for each gene. Partial and full-length sequences were acquired from overlapping partial sequences obtained with forward and reverse primers. Nucleotide sequences are assigned Genbank accession numbers CY067667 to CY067682 and CY096621 to CY096724. RNA extracts from original swab samples negative for virus isolation, were subjected to direct sequencing by the same method described above, in attempt to identify other virus subtypes.

For each genome segment of each virus isolate, BLAST searches at the nucleotide level were initially performed to identify the most closely related viruses. Full-length genome segments from North American and selected Eurasian and South American viruses available at the Influenza Research database (www.fludb.org) were then obtained to be included in the phylogenetic analysis. Sequences of each segment were initially aligned by Clustal V (Lasergene v.8.1.5., DNAStar, Madison, WI) and percent identities were calculated. Sequences from representative isolates were selected and aligned with ClustalW (Lasergene). Rooted phylogenetic trees were generated by Neighbor-Joining method with 1000 bootstrap replicates using PAUP 4.0b10 (Sinauer Associates, Inc., Sunderland, MA).

## Supporting Information

Table S1 Distribution by order and species of wild bird samples collected in Santa Rosa, Jutiapa, and Izabal, 2007–2010.
(DOCX)
