# Supplementary material for: Influenza A Viruses from Wild Birds in Guatemala Belong to the North American Lineage
Source: PLoS One. 2012 Mar 13;7(3):e32873. doi: 10.1371/journal.pone.0032873 (PMC3302778; doi:10.1371/journal.pone.0032873)
Supplement: Table S1 — Distribution by order and species of wild bird samples collected in Santa Rosa, Jutiapa, and Izabal, 2007–2010. (DOCX) [file pone.0032873.s001.docx]

Table S1. Distribution by order and species of wild bird samples collected in Santa Rosa, Jutiapa, and Izabal, 2007-2010

| **Order** | **Species** | **Common name** | **Status** | **Sampled birds*** | **rRT-PCR positives (%)** |
| --- | --- | --- | --- | --- | --- |
| Anseriformes | *Anas clypeata* | Northern shoveler | M | 2 |  |
|  | *A. discors* | Blue-winged Teal | M | 234 | 28 (12%) |
|  | *Aythya collaris* | Red-neck Duck | M | 2 |  |
|  | *Dendrocygna autumnalis* | Black-bellied Whistling Duck | R | 1 |  |
|  |  |  |  |  |  |
| Ciconiiformes | *Butorides virescens* | Green Heron | R | 1 |  |
|  |  |  |  |  |  |
| Columbiformes | *Columba sp.* | Pigeon | R | 1 |  |
|  | *C. livia* | Feral Pigeon | R | 1 |  |
|  | *Columbina inca* | Inca Dove | R | 3 |  |
|  | *C. minuta* | Plain-Breasted Ground-Dove | R | 7 |  |
|  | *C. passerina* | Common Ground-Dove | R | 1 |  |
|  | *C. talpacoti* | Ruddy Ground-Dove | R | 63 |  |
|  | *Leptotila plumbeiceps* | Grey-Headed Dove | R | 4 |  |
|  | *L. verreauxi* | White-Tipped Dove | R | 5 |  |
|  | *Patagioenas cayennensis* | Pale-Vented Pigeon | R | 2 |  |
|  | *Zenaida asiatica* | White-Winged Dove | R | 1 |  |
|  | *Zenaida macroura* | Mourning Dove | R | 6 |  |
|  |  |  |  |  |  |
| Coraciiformes | *Ceryle torquata* | Ringed Kingfisher | R | 1 |  |
|  | *Eumomota superciliosa* | Turquoise-Browed Motmot | R | 6 |  |
|  |  |  |  |  |  |
| Cuculiformes | *Crotophaga sulcirostris* | Groove-Billed Ani | R | 29 |  |
|  | *Piaya cayana* | Squirrel Cuckoo | R | 2 |  |
|  |  |  |  |  |  |
| Gruiformes | *Gallinula chloropus* | Common Moorhen | M | 2 |  |
|  | *Laterallus ruber* | Ruddy Crake | R | 2 |  |
|  |  |  |  |  |  |
| Passeriformes | *Calocitta formosa* | White-Throated Magpie-Jay | R | 2 |  |
|  | *Pachyramphus aglaiae* | Rose-Throated Becard | R | 5 |  |
|  | *Euphonia hirundinacea* | Yellow-Throated Euphonia | R | 1 |  |
|  | *Oryzoborus funereus* | Thick-Billed Seedfinch | R | 9 |  |
|  | *Passerina ciris* | Painted Bunting | M | 2 |  |
|  | *P. cyanea* | Indigo Bunting | M | 4 |  |
|  | *Piranga rubra* | Summer Tanager | M | 3 |  |
|  | *Ramphocelus passerinii* | Scarlet-Rumped Tanager | R | 2 |  |
|  | *Saltator atriceps* | Black-Headed Saltator | R | 1 |  |
|  | *S. coerulescens* | Greyish Saltator | R | 1 |  |
|  | *Sporophila aurita* | Variable Seedeater | R | 3 |  |
|  | *S. torqueola* | White-Collared Seedeater | R | 30 |  |
|  | *Thraupis abbas* | Yellow-Winghed Tanager | R | 1 |  |
|  | *T. episcopus* | Blue-Grey Tanager | R | 2 |  |
|  | *Volatinia jacarina* | Blue-Black Grassquit | R | 3 |  |
|  | *Dives dives* | Melodius Backbird | R | 7 |  |
|  | *Icterus galbula* | Baltimore Oriole | M | 8 |  |
|  | *I. gularis* | Altamira Oriole | R | 4 |  |
|  | *I. pectoralis* | Spot-Breasted Oriole | R | 1 |  |
|  | *I. spurius* | Orchard Oriole | M | 1 |  |
|  | *Psarocolius montezuma* | Montezuma Oropendola | R | 1 |  |
|  | *Quiscalus mexicanus* | Great-Tailed Grackle | R | 45 |  |
|  | *Scaphidura oryzivora* | Giant Cowbird | R | 5 |  |
|  | *Dumetella carolinensis* | Grey Catbird | M | 55 |  |
|  | *Catharus ustulatus* | Swainson's Thrush | M | 46 |  |
|  | *Hylocichla mustelina* | Wood Thrush | M | 2 |  |
|  | *Turdus grayi* | Clay-Colored Thrush | R | 154 |  |
|  | *Dendroica magnolia* | Magnolia Warbler | M | 2 |  |
|  | *D. petechia* | Yellow Warbler | M | 14 |  |
|  | *Icteria virens* | Yellow-Breasted Chat | M | 17 |  |
|  | *Seiurus aurocapillus* | Ovenbird | M | 5 |  |
|  | *S. noveboracensis* | Northern Waterthrush | M | 70 |  |
|  | *Setophaga ruticilla* | American Redstart | M | 1 |  |
|  | *Sphyrapicus varius* | Yellos-Bellied Sapsucker | M | 1 |  |
|  | *Campylorhynchus rufinucha* | Rufous-Naped Wren | R | 3 |  |
|  | *C. zonatus* | Band-Backed Wren | R | 2 |  |
|  | *Thryothorus maculipectus* | Spot-Breasted Wren | R | 4 |  |
|  | *T. pleurostictus* | Banded Wren | R | 1 |  |
|  | *Contopus cinereus* | Tropical Pewee | R | 1 |  |
|  | *Elaenia flavogaster* | Yellos-Bellied Elaenia | R | 1 |  |
|  | *Empidonax sp.* | Flycatcher | M | 1 |  |
|  | *E. trailli* | Willow Flycatcher | M | 3 |  |
|  | *Mionectes oleaginus assimilis* | Ochre-Bellied Flycatcher | R | 1 |  |
|  | *Myiarchus crinitus* | Great Crested Flycatcher | M | 1 |  |
|  | *M. nuttingi* | Nutting's Flycatcher | R | 2 |  |
|  | *M. tuberculifer* | Dusky-Capped Flycatcher | R | 1 |  |
|  | *M. tyrannulus* | Brown-Crested Flycatcher | R | 4 | 1 (25%) |
|  | *Myiozetetes similis* | Social Flycatcher | R | 4 |  |
|  | *Pitangus sulphuratus* | Great Kiskadee | R | 14 |  |
|  | *Tyrannus melancholicus* | Tropical Kingbird | R | 6 |  |
|  | *Vireo griseus* | White-Eyed Vireo | M | 2 |  |
|  | *V. olivaceus* | Red-Eyed Vireo | M | 2 |  |
|  |  |  |  |  |  |
| Psittaciformes | *Aratinga astec* | Aztec Parakeet | R | 2 |  |
|  |  |  |  |  |  |
| Pelecaniformes | *Phalacrocorax auritus* | Double-crested Cormoran | R | 1 |  |
|  |  |  |  |  |  |
| Piciformes | *Melanerpes aurifrons* | Golden-Fronted Woodpecker | R | 23 | 1 (4.3%) |
|  |  |  |  |  |  |
| Strigiformes | *Glaucidium brasilianum* | Ferruginous Pygmy-Owl | R | 6 |  |
| **TOTAL** |  |  |  | **969** | **30 (3.1%)** |

*Cloacal and tracheal swab samples were taken from each individual, with few exceptions depending on the size of the specimens, when the bird was too small only cloacal swab was taken. M: Migratory, R: Resident.
